# Distribution of the Fittest Individuals and the Rate of Muller's Ratchet in a Model with Overlapping Generations

**DOI:** 10.1371/journal.pcbi.1003303

**Published:** 2013-11-07

**Authors:** Jakob J. Metzger, Stephan Eule

**Affiliations:** 1 Max Planck Institute for Dynamics and Self-Organization (MPIDS), Göttingen, Germany; 2 Institute for Nonlinear Dynamics, Department of Physics, University of Göttingen, Göttingen, Germany; University of Texas at Austin, United States of America

## Abstract

Muller's ratchet is a paradigmatic model for the accumulation of deleterious mutations in a population of finite size. A click of the ratchet occurs when all individuals with the least number of deleterious mutations are lost irreversibly due to a stochastic fluctuation. In spite of the simplicity of the model, a quantitative understanding of the process remains an open challenge. In contrast to previous works, we here study a Moran model of the ratchet with overlapping generations. Employing an approximation which describes the fittest individuals as one class and the rest as a second class, we obtain closed analytical expressions of the ratchet rate in the rare clicking regime. As a click in this regime is caused by a rare, large fluctuation from a metastable state, we do not resort to a diffusion approximation but apply an approximation scheme which is especially well suited to describe extinction events from metastable states. This method also allows for a derivation of expressions for the quasi-stationary distribution of the fittest class. Additionally, we confirm numerically that the formulation with overlapping generations leads to the same results as the diffusion approximation and the corresponding Wright-Fisher model with non-overlapping generations.

## Author Summary

Muller's ratchet is a paradigmatic model in population genetics which describes the fixation of a deleterious mutation in a population of finite size due to an unfortunate stochastic fluctuation. Obtaining quantitative predictions of the ratchet rate, i.e. the frequency with which such a mutation fixes, is believed to be important for understanding a broad range of effects ranging from the degeneration of the Y-chromosome to the evolution of sex as a means of avoiding the fixation of deleterious mutations. To obtain a better understanding of how Muller's ratchet operates, we have considered a model with overlapping generations, which allows for the application of methods specifically tailored for the analysis of rare stochastic fluctuations which drive the ratchet. We obtain concise and accurate results for the rate of Muller's ratchet. Additionally, we are able to predict the full distribution of the frequency of the fittest individuals, a quantity of central interest in understanding the ratchet rate and possibly experimentally much more accessible than the rate, in particular when the ratchet rate is very large.

## Introduction

In an asexual population of finite size, weakly deleterious mutations can fix by chance. This phenomenon is due to stochastic fluctuations originating from the finiteness of the population, which can lead to a loss of the fittest class of individuals. If one assumes that the mutation rate does not scale with the length of the genome and that the genome is very long, back mutations are unlikely and can be ignored. In this case the fittest class is lost forever and the number of fixed deleterious mutations increases irreversibly. This process has been termed Muller's ratchet [1], [2] and has been observed experimentally in several studies [3]–[9]. Furthermore, it has been thought to account for the degeneration of non-recombining parts of sexually reproducing organisms such as the Y-chromosome [10] and mitochondrial DNA [6]. Muller's ratchet can also be used to explain the absence of long-lived asexual lineages [11]. Since in the absence of back mutations mutation-free genomes can only be recreated by recombination between mutation-loaded classes, Muller's ratchet provides an appealing explanation for the evolution of sex [12], .

Each time the least-loaded class, i.e. the class with the fewest number of deleterious mutations, is lost, it is said that Muller's ratchet has clicked. Since the rate of the ratchet determines the speed of degeneration of the population, this quantity is of central interest. In its simplest form the rate of Muller's ratchet depends only on the selection coefficient 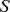, the mutation rate 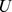 and the size 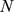 of the population, where it is assumed that each mutation has the same effect so individuals with 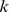 mutations have fitness 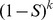. In this case the fitness space is equivalent to an axis counting the number of deleterious mutations and the population can be organized into discrete classes labeled by the number of mutations they carry. The deleterious mutations have the effect of shifting the population to higher values of 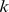. Since the fitness of the respective classes is given by 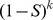, selection works into the opposite direction. In the limit of an infinitely large population these two opposing forces lead to a steady state distribution whose precise form was found by Haigh [14].

If finite populations are considered, however, the calculation of the rate of Muller's ratchet turns out to be an intricate problem, despite its simple formulation. The difficulty arises due to the complex interaction of the fluctuation of the least-loaded class with the rest of the distribution. A detailed quantitative understanding of the behavior of the occupation of the class with the fewest mutations, however, is necessary to determine the mean time to extinction of this class, i.e. the inverse of the ratchet rate. Despite of considerable efforts and recent advances [15]–[23], a quantitative understanding of the ratchet rate remains a challenging open problem.

In its standard form Muller's ratchet was first quantitatively described by Haigh who analyzed a classical Wright-Fisher model of an asexually reproducing population of fixed size 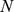. He pointed out that the most important quantity of the ratchet is the average number of individuals in the least loaded class, 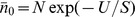, because fluctuations of 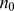 ultimately lead to a click of the ratchet. Haigh also suggested an expression for the rate of the ratchet by fitting to numerical simulations. Gordo and Charlesworth were subsequently able to derive an expression for the ratchet rate by studying deviations from the deterministic equilibrium distribution to obtain approximate expressions for drift and diffusion coefficients, from which they obtained an expression for the ratchet rate that has to be evaluated numerically [18]. Later, again using a diffusion approximation and non-overlapping generations, it was shown by Jain that the ratchet rate cannot depend only on 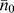 but rather has to depend on 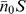 [21]. If 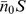 is small, the ratchet clicks frequently and the populations behaves like a wave in 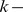
space propagating towards higher values of 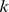. The traveling wave approach to Muller's ratchet was discussed in [24] and provides an appealing quantitative theory for frequently clicking ratchets.

While this regime of Muller's ratchet is relatively well understood, a quantitative understanding of the opposite case 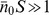 of a rarely clicking ratchet is still lacking and has recently attracted a lot of attention [21], [23]. In this regime the rate of the ratchet is exponentially small in 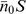 [21] and extinction of the fittest class occurs as the result of a rare, large fluctuation. In contrast to the fast clicking regime the distribution of the population equilibrates to a metastable state after each click. A wide-spread approach in this regime is therefore to consider only the fittest class and apply a phenomenological model for all the classes 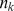, 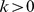, with more mutations than the least-loaded class. Such an approach leads to a one-dimensional approximation where just the fittest class is taken into account. Generally the rate of the ratchet can then be calculated by means of a diffusion approximation as the result of a one-dimensional mean-first passage problem. Recently it was shown how this approach can be improved by accounting for the interaction of the fluctuations of the fittest class with the tail of smaller fitness which can lead to a delayed feedback [23].

Up to now a quantitative treatment of Muller's ratchet relied either on Haigh's model or on the corresponding diffusion approximation. To our knowledge a Moran formulation with overlapping generations has not been employed so far. This is not surprising as in the diffusive limit any quantity should become independent of the respective microscopic formulation and a Moran formulation of Muller's ratchet is expected to be computationally disadvantageous. A Moran formulation, however, can also lead to interesting new approaches to tackle the problem of the ratchet rate analytically.

In the present work we investigate a Moran formulation of Muller's ratchet and show how this model can be approximated by a one-dimensional Moran-process in the regime 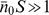 where the ratchet clicks infrequently. We show that this model allows for an analytical solution for the ratchet rate that agrees almost perfectly with values obtained by numerical simulations of the full ratchet. Furthermore, by employing a recently developed method to treat rare, large fluctuations in stochastic population dynamics, we find analytical expressions for the ratchet rate and the quasi-stationary distribution of the fittest class in the parameter range 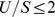, which also agree very well with the corresponding results of the full ratchet. Finally, we confirm numerically that the formulation with overlapping generations leads to the same results as the diffusion approximation and the corresponding Wright-Fisher model with non-overlapping generations.

## Models

In the standard formulation of Muller's ratchet, as considered by Haigh [14], mutations in a population of fixed size 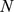 occur at rate 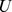 and individuals are classified into different groups according to the number of deleterious mutations they carry, 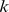. Each mutation reduces the fitness of the genotype 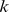 by an amount 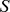 such that the growth rate of an individual with 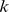 mutations is proportional to 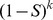. The ratio of the mutation rate 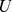 to the mutation effect 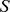, which is denoted by 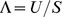, plays a central role in the analysis of the ratchet. Observe that the Haigh model assumes the simplest case of a multiplicative, permutation invariant fitness landscape. An extension to more complicated fitness landscapes with epistatic interactions was discussed in [21].

The reproduction model usually employed in the analysis of Muller's ratchet is Wright-Fisher sampling. It consists of, at each time step, replacing the whole generation of individuals by a multinomial resampling of the current generation [25] weighted by the fitness of the different classes. Thus, according to Haigh [14], if 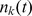 is the number of individuals in generation t which carry 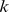 mutations and 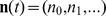, then the distribution of 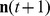 is multinomial with parameters 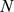 and 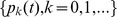, where
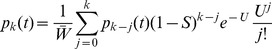
(1)and the mean fitness 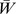 is given by 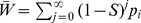.

Wright-Fisher sampling has the advantage of being very efficient for numerical simulations. The downside of the model is, however, that it does not easily allow for analytical methods to be used. Therefore, the corresponding diffusion approximation of the microscopic Wright-Fisher formulation is usually used to predict the click rates of the ratchet.

The second widely applied reproduction model in population genetics is the Moran process, which in contrast to the Wright-Fisher formulation assumes overlapping generations. The Moran process, which we focus on in this article, is amenable to a wider range of analytical methods (at the cost of being slower in numerical simulations) [26]. It is a stochastic process in which at each time step one individual is chosen for reproduction and one for removal from the population. The choice of the individual that reproduces is random, but (similarly to the Wright-Fisher formulation) weighted by the fitness of the class the individual is chosen from. The probability of removal (or death) of an individual is independent of the fitness. Applied to Muller's ratchet this therefore embodies the following procedure: An individual with 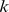 mutations is chosen according to the abundance and selection preference of the class 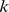 with weight 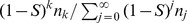. This individual spawns one offspring with 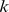 mutations that can then mutate to 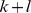 mutations with probability 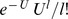. The probability to mutate is thus 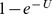, which is the same as in the Wright-Fisher model. Also, one individual with 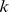 mutations is chosen for removal with probability 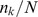 (this may be the one that reproduced). Since on average every individual is chosen for removal once every 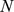 time steps, it is natural to define one generation in the Moran model as 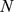 time steps. In all figures, the ratchet click times are thus expressed in generations.

Although different on the microscopic scale, both Wright-Fisher and Moran models usually converge to the same mesoscopic diffusion regime when 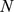 is large and fitness advantages and mutation rates are of order 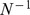. In this limit, the equation describing the evolution of the population is given by
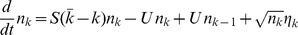
(2)where 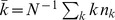 [23]. The uncorrelated Gaussian white noise 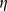 with 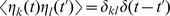 models the stochastic fluctuations due to the finiteness of the population (genetic drift). In the infinite population limit, this equation becomes deterministic and has the steady state solution 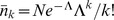 [14]. Also, a time dependent solution of the deterministic model has been obtained [20]. In this paper, we solve Eq. (2) numerically using stochastic Runge-Kutta methods [27].

## Results/Discussion

### Approximate one-dimensional Moran model of Muller's ratchet

**Figure 1:**
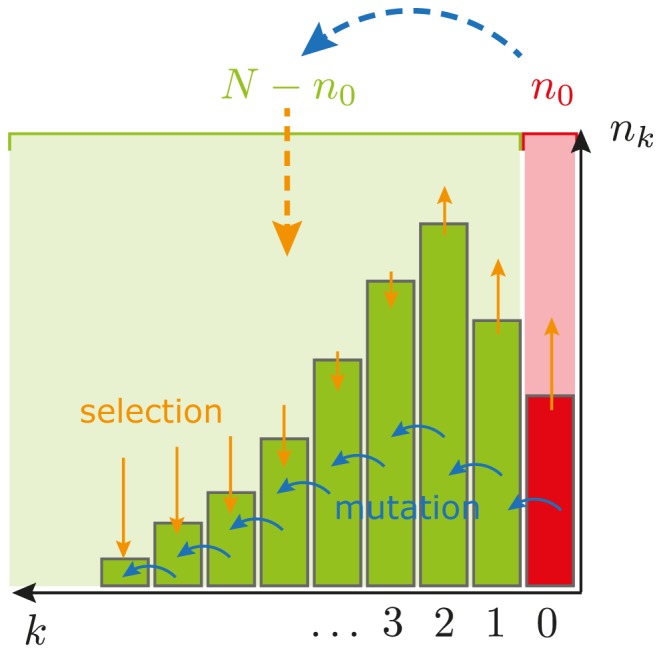
Illustration of Muller's ratchet in the space of deleterious mutations. Individuals are grouped into different classes depending on the number of mutations 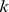 they carry. Mutation (blue arrows) drives the population to higher values of 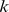, while selection (yellow arrows) opposes this motion, leading to a quasi-stationary distribution (which becomes stationary only in the limit of an infinitely large population). The two-class approximation amounts to putting all mutated individuals in one mutated class (light green box). Both mutation into this class and selection pressure operating on it (large arrows) have to be calculated from the original mutation rates and selection strengths. Since the total population size is conserved, calculating the distribution of the number of individuals in the two classes reduces to the analysis of 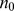 and thus to a one-dimensional problem.

A mathematical analysis of the Moran model for Muller's ratchet is complicated and even the formulation of the corresponding Markov chain [28] is involved and rarely leads to new insights. The important advantage of Moran models, however, is that they can be formulated in terms of a master equation which is a first-order differential equation describing the time-evolution of the probability of a system to occupy each of a number of states [29]. Many methods have been developed to analyze master equations analytically and therefore Moran models are analytically tractable even beyond the diffusion approximation, if only two species are considered. Thus an appealing approach to the analysis of the rate of Muller's ratchet is to approximate the full ratchet by a model consisting only of two species, see Fig. 1. Since we are interested in the loss of the fittest class with zero mutations a natural choice is to consider individuals with zero mutations as one species, and to combine all others in a class which contains all individuals with one or more mutations which in this approximation all have the same fitness 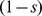 where 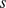 has to be adjusted to account for the actual fitness distribution of the full ratchet model. We discuss this non-trivial approximation in detail below. The constraint of a fixed population size 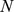 then leads to a one-dimensional model since it is sufficient to consider only the dynamics of the fittest class.

Since in the Moran model the number of individuals can only change by one without further approximations the master equation for the probability 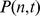 to find 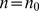 individuals in the fittest class is
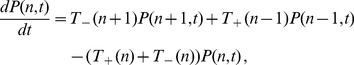
(3)with the transition rates
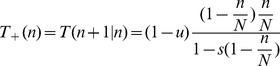
(4)

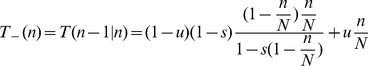
(5)where 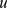 is the mutation rate away from the fittest class and the boundary conditions 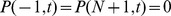 are imposed [25]. Unless specified otherwise, the initial condition is chosen to be concentrated on the equilibrium value of 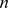 for large 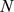 (see below for details). The biological significance of the terms in the equations above are as follows. The probability for one individual of the fittest class to be chosen for birth or death is 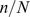. For the mutated class, the probability to be chosen for birth is 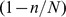 multiplied with the selection disadvantage 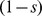, and 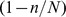 to be chosen to die. The probability for an offspring to mutate is 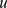, and 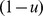 not to mutate. The denominators are normalization factors. Note that we apply the convention of the Moran process where the mutation is divorced from the birth/death process [25]. Here and in the following lowercase letters denote the parameter values of the approximate two-class model, while capital letters denote the parameters of the full ratchet (see also Table 1 for a list of symbols). It is important to note that 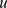 and 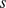 are effective parameters which need to be related to the biologically relevant parameters 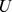 and 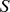. The idea of representing all classes but the fittest as one class was first introduced in [22] for a Wright-Fisher model of Muller's ratchet.

**Table 1:** List of symbols.

Symbol	Description
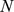	population size
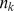 , 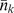	number of individuals with 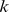 mutations, average (steady state) value
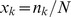	population frequency of class 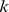
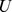 , 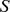	mutation rate and selection coefficient of the full ratchet model
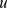 , 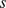	mutation rate and selection coefficient of the reduced two-class model
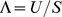 , 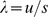	rescaled mutation rate of the full and the two-class model
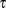	mean click time of the ratchet, i.e. the inverse ratchet rate
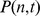	probability to find 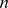 individuals in the fittest class
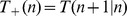	transition rate for increasing the population of the fittest class by one individual
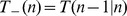	transition rate for decreasing the population of the fittest class by one individual
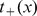 , 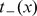	transition rates as a function of the population frequency
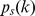	steady state distribution for the probability of an individual to have 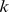 mutations
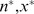	average number of individuals in the fittest class in the two-class ratchet, frequency
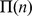 , 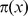	quasi-stationary distribution of fittest class as function of population and frequency
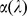 , 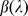	analytically determined prefactor and exponential factor of the ratchet rate

A crucial step in the reduction of the full model of Muller's ratchet to the one-dimensional formulation is the relation of the two mutation rates and fitness disadvantages in the respective models. This mapping is not unique and two reasonable assumptions have to be invoked to relate the two parameters pairs. A plausible approach is to compare the steady state distributions in the infinite population limit of the respective models. For the full ratchet whose dynamics is given by Eq. (1) the well-known steady state distribution for the probability of an individual to have 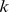 mutations is 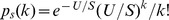. A non-zero steady state of the fittest class in the two-class system can only be obtained in the parameter regime 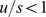 and is given by 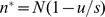. To relate the parameters we now demand that 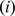 the mean fitness of the full population and 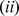 the mean fitness of all individuals carrying a mutation is equal in both models. The mean fitness of the full population in the steady state of the full ratchet is 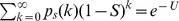 while the mean fitness of all individuals in the two state model is 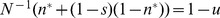. Condition (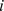) accordingly suggests the relation
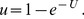
(6)The mean fitness of all individuals carrying mutations is in the full ratchet model given by 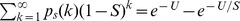. In the two state model this corresponds to 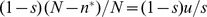. Employing condition 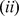 consequently yields the relation
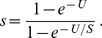
(7)We can also introduce the parameter 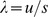 which is related to 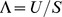 according to
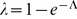
(8)Relation (8) shows that the restriction 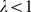 of the two-class model does not restrict the range of 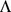.

Before we present the analytical solution for the ratchet rate of this model, let us shortly discuss the validity of the approximation used. To correlate the parameters of the full ratchet and the two state model, we have related properties of the equilibrium solution of an infinite population in both models. This certainly makes sense as long as the typical time 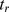 that it takes for the population to relax to a metastable state after each click is much smaller than the mean time 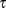 between two successive clicks. This condition is fulfilled in the case of the slowly clicking ratchet, which is the regime we focus on in this work. If the ratchet clicks rapidly the population does not equilibrate after a click and relating the parameters based on equilibrium distributions is clearly not valid.

In the Haigh model mutations are Poisson distributed. It follows that the mutation rate out of the fittest class is 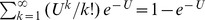. Consequently, from Eq. (6), the mutation rates out of the fittest class are equal in both models which certainly is a reasonable assumption. We note that the occupancy of the fittest class and therefore the rate at which the ratchet clicks is the result of a complicated interplay of all fitness classes. Thus, although the mutation rate out of the fittest class is the same in both models, their rates will differ due to the different fitness distributions.

Furthermore, our second relation (7) entails that the number of individuals which are not in the fittest class is the same in the equilibrium states of both models. Consequently the same holds true for the number of individuals in the fittest class, i.e. 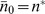. Thus, although the parameter mapping is not unique, it is hard to think of any other relation in the slowly clicking regime as this would consequently violate the properties specified above. Furthermore, our relations (6) and (7) are the same as the expressions previously obtained by Waxman and Loewe [22]. It is important to keep in mind that the parameter mapping is only valid in the rare clicking regime and that other mappings might be more appropriate in the fast clicking regime [22].

#### Exact solution and comparison with full Moran ratchet

With the reduction to a two-class problem as given in the previous section, we can now exploit the advantages that the Moran formulation offers for analytical calculations. The mean click time of the ratchet is given by the mean first time of the population with no mutations reaching zero. It is well known that a solution of such a mean first passage time problem in a two-class model can be formally written as a product of the transitions rates (4) and (5) and is given by [29]

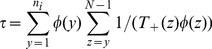
(9)where 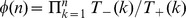 and 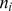 is the initial number of individuals in the lowest mutation class population. We use the initial condition 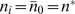. This expression can be evaluated for moderate 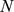, but the number of terms grows quickly with 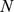 which makes it more and more difficult to evaluate 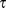.

**Figure 2:**
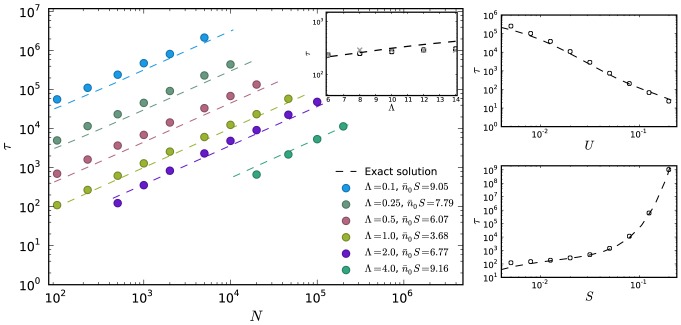
Comparison of analytical and numerical mean ratchet click times. The analytical expression of the mean click time (i.e. the inverse ratchet rate) for the two-class model is compared with numerical simulations of the full ratchet (circles) in the slow ratchet regime. Different colors correspond to different effective mutation rates 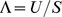 and different 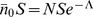. The inset shows that the two-state approximation is still accurate for large 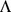, but begins to deviate from the numerical results (parameters: 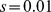, 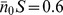). To ensure consistency, we here also compare with the numerical simulations of Neher and Shraiman [23], indicated as crosses. In the small panels, the mean click time is shown for 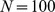 as a function of 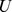 (with 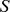 fixed at 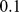) and 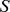 (with 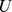 fixed at 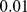), respectively. The range of 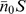 is 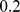 to 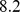 in the upper, and 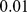 to 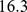 in the lower panel.

To compare our analytical results to the full ratchet we have performed extensive numerical simulations of the full Moran ratchet using the rules detailed in the previous section. We organize our results as follows: The parameters 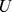 and 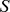 are grouped according to the conditions specified below, and then 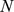 is varied. Since the selection penalty 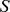 can be interpreted as a timescale [23], we group parameters with the same rescaled mutation rate 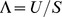. Similarly, since 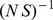 can be interpreted as rescaled variance of the stochastic effects [23], we also group parameters with the same 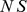, which is then equivalent to keeping 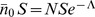 fixed. The corresponding two-class parameters are rescaled as given by (6) and (7). A comparison of the analytical results and the simulations is given in Fig. 2. We observe excellent agreement of the analytic result given by Eq. (9) for the two-class model with the simulation of the full ratchet in the slow ratchet regime, where the two-class approximation is valid. We note that for large 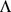 the two-class approximation is still good, however, deviations begin to appear. We attribute this to the fact that the fitness distribution in 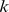-space becomes very broad for large 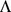 and that therefore the approximation of averaging all mutated individuals into one class becomes less and less accurate.

### WKB-approximation of the ratchet rate

The expression (9) for the mean time to extinction is exact. It gets, however, unwieldy and impractical when larger population sizes are considered. Furthermore, it does not allow for any analytical statements about the distribution of the frequency of the fittest class. Therefore, we want to gain quantitative insight into the ratchet rate and the distribution of the frequency of the fittest class by an approximate treatment of Eq. (3). The most widely applied approach certainly is the diffusion approximation from which by standard methods the mean time to extinction (MTE) 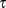 can be calculated analytically [29]. The resulting expression usually has to be evaluated numerically. While the diffusion approximation provides faithful results in the regime where an extinction event is the outcome of a typical fluctuation of the process, it in general may fail to describe the MTE correctly when extinction occurs as the result of a rare, large fluctuation [30]–[32]. In the rare clicking regime the relaxation time to the metastable state is much shorter than the mean time between the clicks and the population equilibrates after each click. It is important to note that in such a scenario the click of the ratchet is due to a rare, large fluctuation away from the metastable state.

An approach to the treatment of master equations which is especially well suited to account for rare event statistics is the WKB- (Wentzel-Kramers-Brillouin) theory. This approximation scheme which is sometimes referred to as the eikonal approximation was first developed for a semi-classical treatment of quantum mechanics and has recently attracted a lot of attention in the context of stochastic population dynamics [33]–[36]. Similar to the diffusion approximation, it replaces the master equation of the Moran process by an analytically tractable equation which in addition allows for a mathematically controlled approximation in terms of powers of the inverse population size. Recently the WKB-approximation has also found its way into evolutionary modeling [30], [37]–[39]. The approach we apply in the following was first considered in [40] and later considerably extended and generalized in [37].

The basic idea relies on the fact that the process can be characterized by a metastable state around which the frequency of the fittest class resides. After a long average time 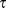 the fittest class is eventually lost and Muller's ratchet clicks. For the approach to work two crucial assumptions have to be made. First, the population size has to be finite and not too small, i.e. 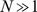. Second the typical relaxation time 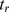 to the metastable state should be much shorter than the MTE, i.e. 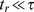. We note that here this condition has to hold anyway in order for the two state approximation to be meaningful. It can be shown that the metastable state, which is sharply peaked around 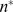, is encoded in the first excited eigenvector 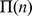 of the master equation (3) which has not decayed at a time 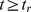 [37]. Thus the shape of the PDF of the metastable state, which is referred to as the quasi-stationary distribution (QSD), is given by 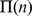. Furthermore, the decay rate of this distribution, i.e. the ratchet rate 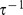, is determined by the first non-zero, positive eigenvalue of the master equation (3). As was shown in [41], the decay of the QSD for times 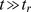 can therefore be obtained as
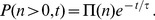
(10)Accordingly, the click probability distribution behaves as
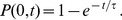
(11)Using Eqs.(3), (10) and (11) the click rate is given by
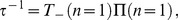
(12)which is just the probability flux into the absorbing state 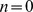.

In the remainder of this section we present an approach to calculate the QSD 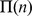 based on a WKB-type approximation. Before employing the WKB ansatz we insert (10) into (3) to obtain, after introducing 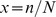,
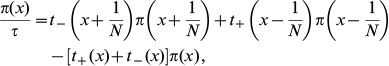
(13)where 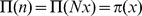 and 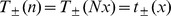. Since we consider the rare-clicking regime of the ratchet, the term on the left-hand side is exponentially small in 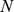 and we can neglect it. The resulting quasi-stationary master equations reads
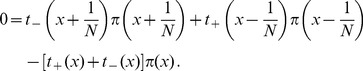
(14)Now we are ready to employ the WKB approach by expressing the solution of this equation by the ansatz [40]

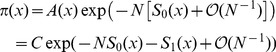
(15)where both 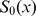 and 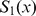 are assumed to be of order unity and 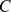 is a normalization constant. Inserting this ansatz into (14), expanding 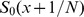 around 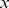 to first order and neglecting terms of order 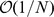, we obtain in leading order
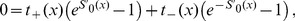
(16)where 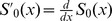. The solution of this equation is given by
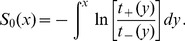
(17)After insertion of 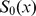 into the ansatz (15) the lowest order solution for the QSD is obtained up to the 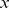-independent normalization constant 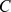. To determine 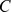 one exploits the fact that the QSD is strongly peaked around 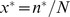 and then assumes it to be of Gaussian shape centered at 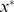 which is normalized to unity. Around the maximum 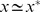 this leads to an approximation of the QSD by 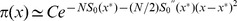 whose normalization yields 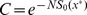. Hence in leading order we obtain for the QSD
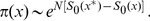
(18)Using this expression of the QSD we can calculate using Eq. (12) the leading order behavior of the click rate
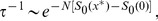
(19)where we have used that 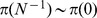 and 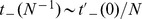 for large 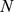. In leading order we thus obtain the anticipated exponential dependence of the ratchet on 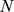 in the rare clicking regime. These results are valid as long as 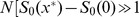 because the WKB-ansatz requires the ratchet rate to be exponentially small. Furthermore, the normalization procedure can be expected to fail if the metastable state is close to the boundary 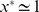 because the Gaussian approximation does not hold anymore.

So far we have obtained the ratchet rate to exponential accuracy only. The next order 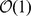-corrections of the WKB-approximation provide the pre-factor of the QSD. They are obtained by expanding 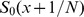 to second order and 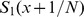 to first order around 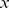. The calculation of the sub-leading corrections is more involved and shall not be carried out in detail here. The crucial step in the calculation is to note that the WKB-solution in leading order is not valid close to the absorbing state at 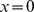. Therefore, the WKB solution has to be matched with an exact recursion solution of the quasi-stationary master equation (14). A detailed account of this method is given in [37]. Following the steps in [37] we obtain for the QSD
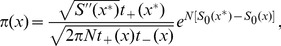
(20)where the transition rates can be obtained from Eqs. (4) and (5) and 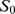 is given by Eq.(17).

The WKB solution for the inverse of the ratchet rate is given by
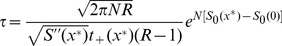
(21)with 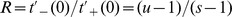.

**Figure 3:**
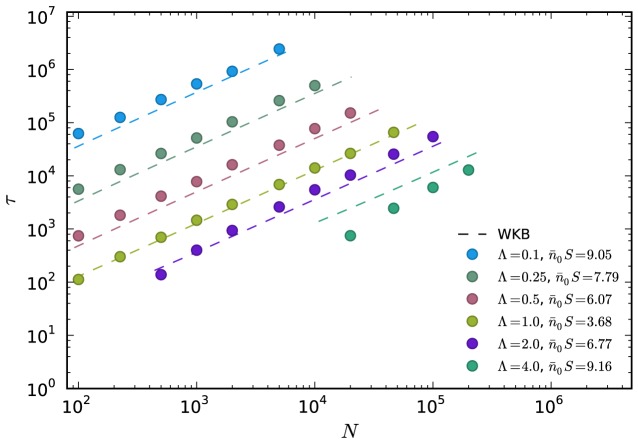
Comparison of the simplified WKB-solution (23) for the inverse ratchet rate and the numerical simulations of the full ratchet. Different colors correspond to different effective mutation rates 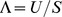 and different 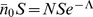. Deviations from the full WKB result, Eq. (22), only occur at small 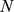, see also supporting Fig. S1.

Inserting the respective transition rates, we obtain for the mean time to extinction of the fittest class, i.e. the inverse of the ratchet rate
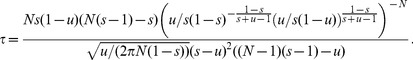
(22)This expression provides an exact result to order 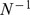. To gain a deeper understanding of the WKB-solution one can simplify the unwieldy expression (22) for 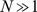. Keeping 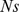 and 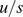 constant and expanding in 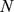, we obtain to leading order in 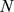 the approximation
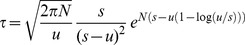
(23)which is almost indistinguishable from the WKB-solution (22) for 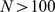. In Fig. 3 we have compared this result for different parameters to the numerical results of the full ratchet. The WKB approximation of the mean time to extinction in the two state model agrees in the range 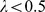 corresponding to 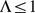 almost perfectly with the numerical results of the full ratchet. While the WKB-prediction is still quite good for 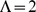 it starts to deviate for increasing values of 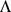. The parameter range in which the WKB-theory works thus is more restricted than in the two-class model. This can be explained by noting that for 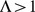 the two-class approximation is still valid if the ratchet operates in the rare clicking regime, i.e. if 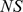 is chosen to be large enough, see Fig. 2. The WKB-theory on the other hand breaks down if 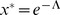 is close to the absorbing state at 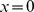 independent of 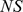. A comparison of the exact solution (9), the WKB-solution (22) and the approximation (23) is provided as supporting information (Fig. S1).

**Figure 4:**
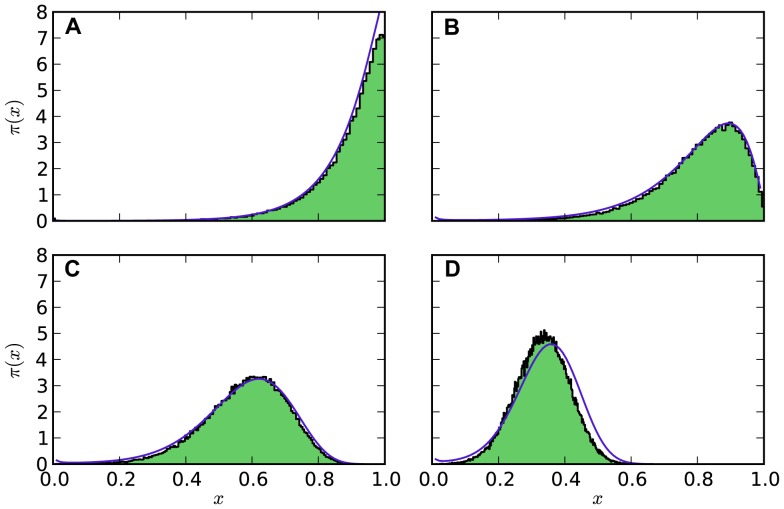
Distribution of individuals in the fittest class. Comparison of the WKB-solution (20) for the quasi-stationary distribution 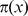 (blue line) with the distribution of the fittest class of the full ratchet obtained by numerical simulations for 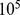 realizations (green histogram) at 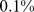 of the respective click times. At this time, the distribution of the class with the lowest number of mutations has already relaxed to the quasi-stationary state, while in almost no realization a click has already occurred. The parameters used are (Panel A) 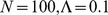, (Panel B) 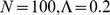, (Panel C) 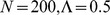, (Panel D) 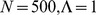, and in all cases 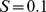. The analytic solution of the two-class model fits the numerical distribution obtained for the full ratchet very well. Deviations occur when the fixed point of the deterministic solution, 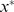, begins to approach the absorbing point at 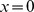, which is where the WKB approximation is expected to break down.

The WKB-theory not only yields results for the ratchet rate but is also capable of describing the frequency distribution of the fittest class in the metastable state, i.e. the QSD, because the parameters of the two-class model were chosen such that the size of the fittest classes match in both models. One can therefore also expect that the QSD of the fittest class is approximately the same in both models. In Fig. 4 we have compared the numerically obtained size of the fittest class in the full ratchet model with the WKB-solution (20) and observe a striking agreement. As anticipated, the WKB-theory starts to deviate if the deterministic fixed point 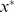 is close to the absorbing point at 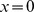 and if 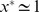.

### Comparison of Moran, Wright-Fisher and diffusion models

Let us now discuss how the presented analysis is related to previous studies on the rate of Muller's ratchet. Preceding works have mostly considered the diffusion approximation in the form of the stochastic differential equation (2) to approach Muller's ratchet analytically, while numerical simulations have relied on Haigh's model with non-overlapping generations using Wright-Fisher sampling, Eq. (1). For this reason it is first of all necessary to check that the Moran model of the full ratchet yields the same rates as the Wright-Fisher model and the diffusion approximation.

We note that some care has to be taken to ensure that the diffusive limit of the Wright-Fisher model has the same diffusion constant as the corresponding Moran formulation, since these usually differ by a factor of two [28]. Since fluctuations scale with 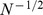, one possibility to take this into account is to consider the Wright-Fisher model with 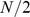 individuals, which is what we do in the simulations presented below.

**Figure 5:**
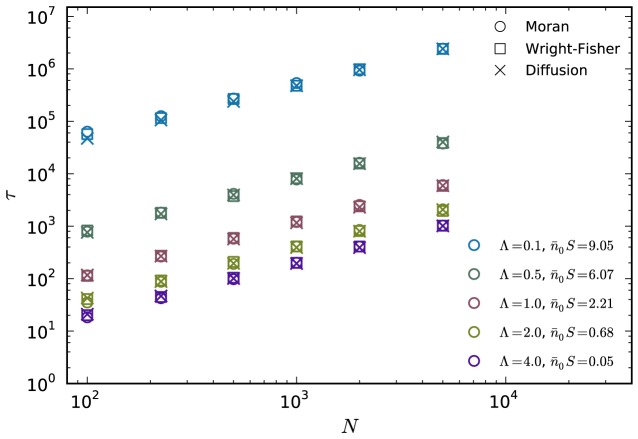
Comparison of different models. Average click times 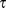 of Muller's ratchet for the Moran model (circles), Wright-Fisher model (squares) and the diffusion limit of the two models (crosses). Same sets of parameters 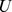 and 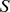 are shown in the same color. We observe perfect coincidence of all three models for both slowly and fast clicking ratchets.

We have performed numerical simulations of the Wright-Fisher model and have numerically integrated the stochastic differential equation (2) using stochastic Runge-Kutta methods. To compare the three different approaches, the click times averaged over 1000 realizations for each model for different values of 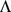 and 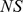 similarly to the previous sections are presented in Fig. 5. We observe excellent agreement of the two macroscopic models and the diffusive description for slow and fast ratchets.

After ensuring that the rate of Muller's ratchet is independent of the microscopic reproduction model, let us now explain why a Moran model is nevertheless essential for the presented approach. The Moran model is exclusive because it can be formulated in terms of a master equation for which well-known analytical methods exist that allow alternatives to the diffusion approximation. The WKB-approximation is one example of these methods that is particularly useful to describe rare, large fluctuations. Most classical and recent works, however, have considered a one-dimensional diffusion approximation to analyze the dynamics of the fittest class and calculated the ratchet rate as the mean time to extinction of this process. Given the fact that the rare, large fluctuations are responsible for the ratchet clicks, a Moran model certainly deserves a detailed analysis.

In order to compare our solution for the click rate Eq. (23) with the preceding works, we rewrite this expression as
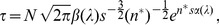
(24)where 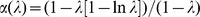 and 
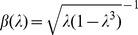
. This expression exhibits the same scaling behavior in the effective parameters 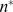, 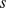 and 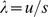 as the one found by Jain in the rare clicking regime with the parameters 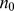, 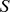 and 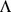 of the full ratchet, who was the first to show that the ratchet rate cannot depend only on 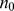 but has to depend on 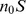 [21]. Thus by using Eqs. (6–8) and noting that 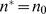 the form of Eq. (24) agrees with findings of Jain for small 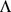 and small 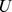. Our solution exhibits 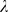-dependent functions in the exponent and the pre-factor which is in contrast to the result of Jain where these factors have to be replaced by a constant 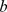 which is sometime referred to as the Haigh factor. Since 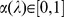 and 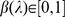 the values of both functions are close to the values between 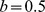 and 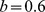 which were ad hoc chosen for this constant. In a recent work, Neher and Shraiman investigated the propagation of fluctuations in the fitness distribution [23]. In the course of their work they also found the Haigh factor to be 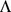-dependent which they could attribute to a time delay between the fluctuations in the fittest class and the fluctuations of the mean fitness, thereby extending the classical work of Haigh [14], Stephan et al. [15], Gordo and Charlesworth [18], and also the more recent work by Jain [21]. While Neher and Shraiman used path integral techniques to obtain an expression similar to Eq. (24), and had to calculate the value of the Haigh factor numerically, our approach determines the ratchet rate including the Haigh-factor analytically, at the cost of being restricted to small 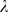. Neher and Shraiman plot their result for 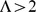, for which the WKB is not a good approximation any more. This and the fact that also 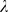 is no longer similar to 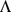 may explain that our result for the Haigh factor decays faster than the numerical results of Neher and Shraiman.

### Summary and conclusions

Muller's ratchet has been proposed as a simple model for the degeneration of asexual populations and non-recombining parts of sexually reproducing populations. The quantitative understanding of the ratchet rate is complicated due to the significant influence of rare, large fluctuations of the number of individuals in the fittest class. This effect is most prominent in the important regime where the ratchet clicks infrequently, which is characterized by a relaxation of the ratchet to a metastable state after each click. The fact that the extinction of the fittest class is due to such a rare, large fluctuation and not the cause of a typical fluctuation prohibits simple diffusive treatments of the ratchet and thus generates difficulties in finding an analytical expression for the ratchet rate.

In this article, we have obtained such an analytical expression by considering a simplified Moran model of Muller's ratchet that reduces the calculation of the ratchet rate to the simpler problem of calculating the mean time to fixation of a deleterious allele. We have shown that in the rare clicking regime the rates predicted by this two-class Moran model agree almost perfectly with the rates of the full ratchet obtained numerically. Furthermore, the formulation of the two-class model in terms of a one-dimensional master equation allows for the application of an approximation scheme which specifically accounts for the effects of rare, large fluctuations. This WKB-theory is a controlled approximation in terms of the inverse population size and provides a closed analytical solution without any free parameters. Our method yields the same scaling form of the ratchet rate as previously obtained by Jain [21]. In contrast to Jain, we find a 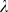-dependent exponential prefactor. This supports the findings of Neher and Shraiman who also suggested a 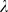-dependence of the “Haigh factor” [23]. While in their work the factor had to be estimated numerically, our theory yields an analytical prediction for this quantity.

Additionally, we have been able to obtain analytical results for the frequency distribution of the fittest class in the metastable state that are in excellent agreement with numerical simulations. This distribution has been alluded to in several of the previous works on Muller's ratchet, but has remained elusive up to now. Our analytical description of the distribution provides a more complete understanding of the ratchet, particularly because the distribution is formed at a fraction of the ratchet click time. Also, the fact that distinguished non-Gaussian tails can be observed in the frequency distribution again emphasizes the necessity to go beyond simple diffusion approximations to describe the ratchet rate analytically.

We have shown that a Moran formulation in conjunction with a reduction to a two-class model and the subsequent application of a WKB-type approximation can provide a viable route for the quantitative prediction of rare, but crucially large fluctuations in simple models of population genetics. We anticipate that models covering additional effects such as epistasis can be included in this framework and, more generally, that the methodology presented here can also be applied in other areas of computational biology where a process is driven by rare stochastic fluctuations.

## Supporting Information

Figure S1
**Comparison of the different analytical results.** Shown are the click times calculated using the exact solution Eq. (9), the WKB approximation Eq. (22), and the simplified WKB expression for large 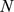, Eq. (23). The WKB results are in excellent agreement with the exact solution for the rare clicking regime when the equilibrium point of the deterministic equation, 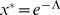, is sufficiently far away from 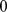. For large 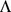, the WKB approximation begins to deviate from the exact solution.(EPS)
